# Albumin-bound paclitaxel combined with lobaplatin using different administration methods in the treatment of cervical cancer: Study on the efficacy and its effect on immune function and inflammatory response

**DOI:** 10.12669/pjms.41.8.11687

**Published:** 2025-08

**Authors:** Runting Li, Ran Song, Ying Jiao, Dan Liu, Libing Zhang

**Affiliations:** 1 Runting Li Department of Gynecology, The NO.2 Hospital of Baoding, Baoding 071000, Hebei, China; 2 Ran Song Department of Gynecology, The NO.2 Hospital of Baoding, Baoding 071000, Hebei, China; 3 Ying Jiao Department of Gynecology, The NO.2 Hospital of Baoding, Baoding 071000, Hebei, China; 4 Dan Liu Department of Gynecology, The NO.2 Hospital of Baoding, Baoding 071000, Hebei, China; 5 Libing Zhang Department of Gynecology, The NO.2 Hospital of Baoding, Baoding 071000, Hebei, China

**Keywords:** Albumin, Cervical cancer, Interventional chemoembolization, Lobaplatin, Paclitaxel

## Abstract

**Objective:**

To investigate the efficacy of the combination therapy of albumin-bound paclitaxel and lobaplatin in the treatment of cervical cancer, and to explore its effects on immune function and inflammatory response.

**Methodology:**

This was a retrospective study. This study enrolled 108 cervical cancer patients admitted to The No.2 Hospital of Baoding from January 2020 to March 2024, and divided them into two groups according to different administration methods. Compared the short-term and long-term efficacy, side effects, immune functions, inflammatory factor levels, etc. between groups.

**Results:**

Comparison of the short-term efficacy between groups showed that the intervention group had a significantly higher response rate (83.33% vs. 42.59%) and disease control rate (92.59% vs. 68.52%) than those in the drip group(P<0.05). The 2nd year survival rate (74.07% vs. 46.30%), 3rd year survival rate (64.81% vs. 29.63%) were significantly higher, and the survival time was obviously longer in the intervention group than those in the drip group (P<0.05). Furthermore, the levels of immune function indicators (Th1 and Th2) and inflammatory factors (TGF-β, IFN-γ and TNF-α) were significantly lower, while IL-10 level was obviously higher in the intervention group after treatment than those before treatment and in the drip group (P<0.05).

**Conclusion:**

Albumin-bound paclitaxel combined with lobaplatin has a significant therapeutic effect for interventional chemoembolization of patients with cervical cancer. It may significantly improve the short-term and long-term clinical efficacy and immune function, and alleviate the inflammatory condition of cervical cancer patients.

## INTRODUCTION

Cervical cancer is a common malignant tumor that threatens the reproductive health of females, especially common in China. Cervical cancer patients are prone to lymph node metastasis, presenting with serious clinical symptoms such as systemic disease, fever, and weight loss.^1^,^2^ Surgery is the primary therapeutic option for the treatment of patients with advanced cervical cancer. Featured by atypical early clinical symptoms, cervical cancer is usually diagnosed in the middle-advanced stage.^3^ Consequently, chemotherapy is adopted for treating cervical cancer to control the condition and prolong tumor control time.

Albumin-bound paclitaxel is a broad-spectrum anti-tumor chemotherapeutic agent that is conjugated with human albumin and paclitaxel. Compared with conventional paclitaxel, it has several advantages such as higher peak plasma concentration, tissue permeability, tissue distribution, tumor tissue targeting, and lower toxicity.^4^ Lobaplatin, a third-generation platinum-based compound, exhibits significant cytotoxicity against various tumor cell lines, with relatively low toxicity to the kidneys and nerves. Acting as a potent anti-cancer agent, lobaplatin has a broad spectrum of biological activity and low toxicity.^5^ Chemotherapy has been recognized to be a common therapy for cervical cancer clinically. In the clinical setting, an individualized chemotherapy regimen can be developed based on factors such as the pathological type, age, and fertility needs of patients with cervical cancer.^6^

Interventional chemoembolization is a novel chemotherapy protocol that can achieve a high concentration of chemotherapeutic agents locally, with high safety and a high clearance rate of small lesions.^7^ Interventional chemoembolization has been applied in the clinical treatment of advanced malignant tumors such as liver cancer and lung cancer, with significant effect. However, there are few studies on its application in cervical cancer.^8^ Accordingly, this retrospective study was performed to compare the difference in efficacy of albumin-bound paclitaxel combined with lobaplatin through intravenous administration and interventional embolization by analyzing the clinical data of 108 cervical cancer patients.

## METHODOLOGY

This was a retrospective study. One hundred and eight patients with cervical cancer who visited The No.2 Hospital of Baoding from January 2020 to March 2024. The follow-up ended in March 2024. The enrolled patients were randomly divided into a drip group and an intervention group using a random number table method (n=54 each group). According to the data of each indicator in the pre-survey, the sample size is estimated by 95% confidence interval, and the largest one is the sample size of the study. The sample size required for each group was ≥50 cases on the basis of Fisher exact probability.

### Ethical approval:

The study was approved by the Institutional Ethics Committee of The No.2 Hospital of Baoding (No.: KY2023005; Dated: January 1, 2023), and written informed consent was obtained from all participants.

### Inclusion criteria:

- Patients who met the diagnostic criteria for cervical cancer in the *White Paper on the Current Status of Cervical Cancer Screening and Early Precision Diagnosis in 2024*.^9^
- Patients with complete clinical data.
- Patients with primary cervical cancer.
- Patients who were conscious and signed informed consent forms.

### Exclusion criteria:

- Patients with severe infection.
- Patients with other major organ dysfunction.
- Patients with hematopoietic disorder and immune dysfunction.
- Patients with mental disorders.
- Breastfeeding and pregnant women.
- Patients allergic to experimental drugs.

### The drip group:

Patients in this group were provided with pre-treatment of 20 mg dexamethasone, 400 mg cimetidine, and 40 mg diphenhydramine via intramuscular injection 30 minutes before intravenous administration. If there was no allergic reaction, patients were given an intravenous drip of 75 mg/m^2^ albumin-bound paclitaxel added to 15 ml 0.9% Sodium Chloride Injection for 30 minutes. Then, patients were injected with Lobaplatin Injection (Registration No: H20050308, Hainan Chang’an International Pharmaceutical Co., Ltd.) mixed with 10mg 250ml of 5% Glucose Injection.

### The intervention group:

Patients in this group were administered through interventional embolization. The puncture cannula was inserted into the iliac artery using the Seldinger method, and a catheter was inserted at the entrance of the common iliac artery. Bilateral iliac artery angiography was performed to examine the uterine artery and tumor vasculature. According to the results of angiography, the catheter was inserted into the uterine artery branch of the internal iliac artery, followed by the injection of a mixed solution of 175 mg/m^2^ Albumin-bound Paclitaxel Injection, 75 mg/m^2^ Lobaplatin Injection, 0.9% Sodium Chloride Injection 10 ml, and 5 ml of Iodinated Oil through the catheter slowly. The embolization was achieved by using an absorbable gelatin sponge. Both groups were given three weeks of treatment, with three consecutive cycles of medication administered on the first day of each treatment cycle.

### Outcome measures:

- *Therapeutic efficacy:* Complete response(CR): Complete remission of patients’ clinical symptoms; partial response (PR): partial remission of patients’ clinical symptoms; stable disease (SD): stable condition of patients; and progressive disease (PD): further deterioration of patients’ clinical symptoms. The response rate= (CR+PR) ÷ total cases×100%, and disease control rate= (CR+PR+SD)÷total cases×100%. All response rate(ORR), Disease control rate(DCR).
- *Immune function indicators:* The levels of immune function indicators such as helper T (Th) cells (Th1, Th2, and Th17) and regulatory T (Treg) cells in patients before and after treatment were detected using flow cytometry.
- *Inflammatory factors:* This study detected inflammatory factors of transforming growth factor-β (TGF-β), interleukin-10 (IL-10), interferon-γ (IFN-γ), and tumor necrosis factor-α (TNF-α). Patients should avoid consuming high-purine or high-protein foods before the examination. Venous blood should be drawn from patients in the morning of the next day after fasting for 10 hours The detection was completed by enzyme-linked immunosorbent assay using corresponding reagent kits purchased from Xiamen Huijia Biotechnology Co., Ltd. (IL-10 Lot No.: EHJ-40290; and IFN-γ Lot No.: EHJ10185) and Shenzhen Biowit Technologies Co., Ltd. (TNF-α Lot No.: DL001396; and TGF-β Lot No.: DL002896).
- *Adverse reactions*: This study observed and recorded the adverse reactions that occurred in both groups.
- \All patients were followed up for three years to record their survival status.

### Statistical analysis:

Data analysis in this study was completed using SPSS22.0. Measurement data that conformed to normal distribution were expressed by () and compared using *t*-test; while categorical variables were described as the number of cases and percentage (n,%), and compared using χ^2^ test. The Kaplan-Meier curve was plotted to analyze the average survival time of the two groups of patients, and the log-rank test to compare the differences between groups. A value of *P*<0.05 was used to indicate a statistically significant difference.

## RESULTS

As shown in Table-I, there was no significant difference in the comparison of general data between the two groups of patients (P>0.05). The response and disease control rates of patients in the intervention group were 83.33% and 92.59%, which were 42.59% and 68.52% in the drip group, respectively. Comparison of short-term therapeutic effects between groups showed that the response and disease control rates of patients in the intervention group were significantly higher than those in the drip group (P<0.05), as shown in Table-II. There was no statistically significant difference in the levels of immune function indicators between the two groups before treatment (P>0.05). Th1 and Th2 levels in the intervention group after treatment were significantly lower than those before treatment and in the drip group (P<0.05; Table-III).

**Table-I:** Comparison of general data.

Groups	n	Age (years)	Tumor diameter (cm)	Staging			Pathological type		
				Ib	IIa	IIIb	Squamous cell carcinoma	Adenocar-cinoma	Adenosquamous carcinoma
Intervention group	54	46.67±3.65	4.67±0.65	10	18	26	28	21	5
Drip group	54	47.34±3.84	4.57±0.45	9	19	26	29	19	6
t/χ² value	-	0.977	1.336	2.321			0.208		
P value	-	0.331	0.184	0.313			0.901		

**Table-II:** Comparison of short-term therapeutic effects between the two groups of patients [n(%)].

Groups	n	CR	PR	SD	PD	Response rate	Disease control rate
Intervention group	54	17 (31.48)	28 (51.85)	5 (9.26)	4 (7.41)	45 (83.33)	50 (92.59)
Drip group	54	13 (24.07)	18 (33.33)	14 (25.93)	9 (16.67)	23 (42.59)	37 (68.52)
X^2^ value	-	-	-	-	-	19.218	9.99
P value	-	-	-	-	-	<0.001	0.002

**Table-III:** Changes in immune function indicators (*χ̅*±*S*).

Groups	Time point	Th1 (%)	Th2 (%)	Treg (%)	Th17 (%)
Intervention group (n=54)	Pre-treatment	7.85±1.17	3.21±0.58	3.54±1.38	4.51±0.79
	Post-treatment	1.52±0.83	1.27±0.23	3.38±1.27	4.27±0.83
	t1 value	37.211	28.821	1.102	2.550
	P1 value	<0.001	<0.001	0.276	0.014
Drip group (n=54)	Pre-treatment	7.87±1.18	3.25±0.54	3.51±1.35	4.52±0.87
	Post-treatment	3.85±0.66	2.57±0.31	3.28±1.37	4.57±0.85
	t1 value	29.369	11.069	1.714	0.533
	P1 value	<0.001	<0.001	0.092	0.596
Pre-treatment inter-group comparison	t2 value	0.088	0.371	0.114	0.063
	P2 value	0.930	0.712	0.909	0.950
Post-treatment inter-group comparison	t3 value	16.149	24.765	0.393	1.856
	P3 value	<0.001	<0.001	0.695	0.066

**Fig.1:**
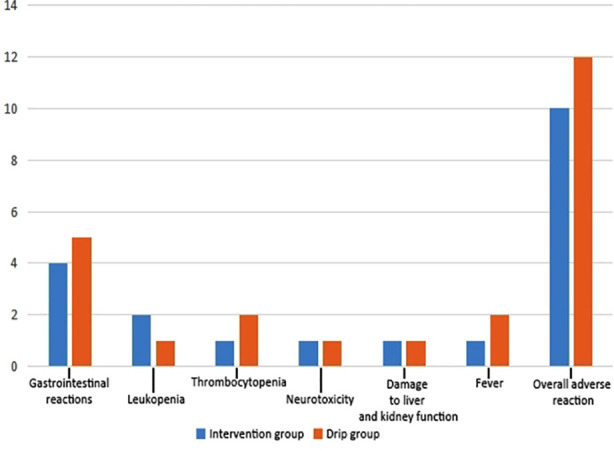
Comparison of adverse reactions between the two groups of patients.

No statistically significant difference was found in the levels of inflammatory factors between the two groups before treatment (P>0.05). The levels of TGF-β, IFN-γ and TNF-α were significantly lower, while the level of IL-10 was obviously higher in the intervention group after treatment than those before treatment and in the drip group (P<0.05; Table IV). The incidence of adverse reactions was 18.25% in the intervention group, and 22.22% in the drip group. No statistically significant difference was detected in the incidence of adverse reactions between groups (χ^2^=0.228, P=0.633; Fig.1).

**Table-IV:** Changes in inflammatory factors (*χ̅*±*S*).

Groups	Time point	TGF-β (nmol/L)	IL-10 (pg/mL)	IFN-γ (pg/mL)	TNF-α (pg/mL)
Intervention group (n=54)	Pre-treatment	27.85±5.17	14.83±2.85	24.61±5.18	651.85±31.17
	Post-treatment	10.22±3.33	21.41±3.72	12.45±2.16	210.22±23.83
	t1 value	30.285	18.936	20.263	136.694
	P1 value	<0.001	<0.001	<0.001	<0.001
Drip group (n=54)	Pre-treatment	28.87±5.18	14.82±2.88	24.65±5.17	658.87±32.18
	Post-treatment	18.55±4.66	16.65±2.61	18.52±3.13	309.55±28.66
	t1 value	15.389	4.985	11.774	99.629
	P1 value	<0.001	<0.001	<0.001	<0.001
Pre-treatment inter-group comparison	t2 value	1.024	0.018	0.040	1.151
	P2 value	0.308	0.986	0.968	0.252
Post-treatment inter-group comparison	t3 value	10.687	7.697	11.727	19.583
	P3 value	<0.001	<0.001	<0.001	<0.001

**Fig.2:**
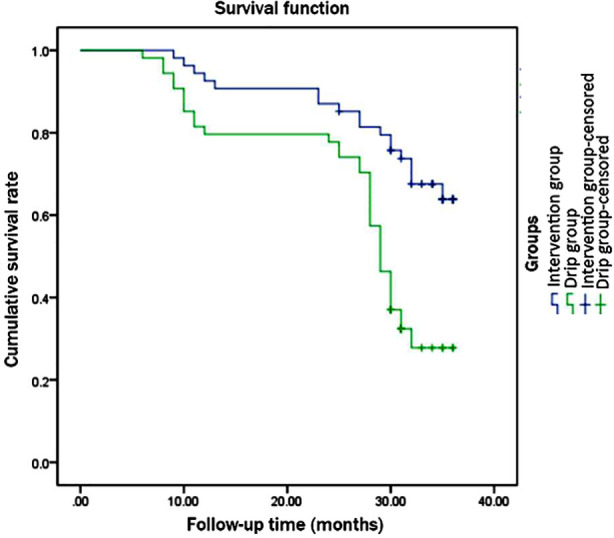
Survival time of the two groups of patients.

As illustrated in Table-V and Fig.2, except for no significant difference in the comparison of the 1st year survival rate between groups, the 2nd year survival rate and 3rd year survival rate were significantly higher, and survival time was obviously longer in the intervention group than those in the drip group (P<0.05).

**Table-V:** Comparison of survival time between the two groups of patients.

Groups	1st year survival rate	2nd year survival rate	3rd year survival rate	Survival time
Intervention group (n=54)	47 (87.04)	40 (74.07)	35 (64.81)	36 (9, 36)
Drip group (n=54)	43 (79.63)	25 (46.30)	16 (29.63)	29 (6, 36)
χ^2^ value	1.067	8.694	13.412	3.152
P value	0.302	0.003	<0.001	0.002

## DISCUSSION

This study used albumin-bound paclitaxel combined with lobaplatin to treat cervical cancer patients. These results support that compared with intravenous administration, albumin-bound paclitaxel combined with lobaplatin for interventional chemoembolization has stronger effects on reducing inflammatory cytokine levels and improving immune function in cervical cancer patients. Simultaneously, the 2nd year survival rate, 3rd year survival rate, and survival time of patients in the intervention group were significantly higher than those in the drip group; besides, no statistically significant difference was observed in the incidence of adverse reactions between groups (*P*>0.05). It may suggest a better long-term efficacy of interventional embolization, without increasing the risk of complications.^10^,^11^

Interventional chemotherapy is a new solution of adjuvant chemotherapy for locally advanced cervical cancer. In this study, patients in the intervention group and drip group received albumin-bound paclitaxel combined with lobaplatin through intervention embolization and intravenous injection, respectively. According to the results, after treatment, the intervention group had significantly better short-term efficacy than that in the drip group; obviously lower levels of Th1 and Th2 (immune function indicators), TGF-β, IFN-γ and TNF-α (pro-inflammatory factors); and evidently higher IL-10 level (anti-inflammatory factor) compared with those before treatment and in the drip group (*P*<0.05). Paclitaxel is currently the most effective drug against cervical cancer. However, to overcome its shortcoming of poor water solubility, co-solvents need to be added as a vehicle during treatment, resulting in many side effects after treatment that compromise the treatment efficacy and reduce the safety.^12^,^13^ While albumin nanoparticles as nanocarriers can quickly disperse in the tissue after injection to enhance tissue affinity, which can bind to Gp60 on the cell membrane, and activate caveolin-1 in the membrane.^14^ Simultaneously, paclitaxel can be delivered to the target site through endothelial cells, and drug-containing nanoparticles are introduced into tumor cells through gaps in capillary walls called intercellular clefts to achieve targeted therapy, thereby achieving more stable and accurate treatment.^15^-^17^

Lobaplatin, one of the front-line third-generation platinum-based anti-tumor agents, has been confirmed to have obvious cytotoxic effects on various tumor cell lines in several animal and human experiments. Lobaplatin can cause damage to tumor cell DNA by forming Pt-GG and Pt-AG intra-strand cross-links. The inter- and intra-strand cross-linking can block DNA replication and transcription within tumor cells, promoting inter- or inter-strand DNA bridge formation, causing DNA distortion, disrupting tumor cell cycles, and inducing tumor cell apoptosis. It is a potent antitumor agent. Considering its advantages of high activity, good water solubility, broad-spectrum anti-tumor activity and low toxicity,^18^-^20^

### Limitations

However, the limitation of this study is a small number of samples, and more indexes should be observed to evaluate the performance of the treatment plan. In view of this, more samples and indexes of pain behaviors, and inflammatory mediators may be investigated in future studies to further validate the findings of this study.

## CONCLUSIONS

Albumin-bound paclitaxel combined with lobaplatin has a significant effect on interventional chemoembolization of cervical cancer. It may significantly improve the short-term and long-term clinical efficacy and immune function, and alleviate the inflammatory condition of cervical cancer patients, without an increase in the probability of complications.

### Authors’ Contributions:

**RL** and **RS:** Carried out the studies, drafted the manuscript, and are responsible and accountable for the accuracy or integrity of the work.

**YJ** and **DL:** Performed the statistical analysis and participated in its design. Critical Review.

**LZ:** Collected the data, performed the analysis, critical review, were involved in the writing of the manuscript.

All authors have read and approved the final manuscript.
